# Geriatric Oncology: Cognition and Communication

**DOI:** 10.1093/geroni/igab046.2579

**Published:** 2021-12-17

**Authors:** Patricia Parker, Yesne Alici, Christian Nelson, Smita Banerjee, Charlotte Malling, Nessa Coyle, Beatriz Korc-Grodzicki

**Affiliations:** 1 Memorial Sloan Kettering Cancer Center, Memorial Sloan Kettering Cancer Center, NYC, New York, United States; 2 Memorial Sloan Kettering Cancer Center, New York, New York, United States; 3 Memorial Sloan Kettering Cancer Center, Memorial Sloan Kettering Cancer Center/New York, New York, United States; 4 Memorial Sloan Kettering Cancer Center, Memorial Sloan Kettering Cancer Center, New York, United States

## Abstract

With the dramatic increase in the older adult population, assessment and care of chronic diseases of aging, notably cancer, cognitive impairment and functional decline, have increasing clinical importance. Most healthcare practitioners (HCPs) receive minimal education in geriatrics and/or communication skills and are not optimally prepared to treat older cancer patients. Geriatric Oncology: Cognition and Communication (Geri-Onc CC) trains HCPs to identify cognitive impairment and/or functional decline and improve communication with patients and caregivers. Geri-Onc CC is a 2-day virtual training. Day 1 covers depression, dementia, delirium, pharmacology, cognitive rehabilitation, language barriers, and decision-making capacity. Day 2 focuses on communication skills experiential practice in geriatric syndromes, cognitive syndromes and shared decision making. In addition, HCPs engage in 6 bimonthly web-based collaborative learning activities post-training. Thus far, three cohorts have participated (n=56). Participants were primarily female (88%), 68% non-Hispanic White, and represented multiple disciplines [psychologists (29%) social workers (25%), physicians (21%), others(25%)] and they work in various settings: comprehensive cancer centers (43%),community hospitals (18%), and others (33%). Most (48%) have been caring for older cancer patients for 1-5 years. All reported the training had value to them as a clinician, increased their knowledge of geriatrics, and helped them meet their training goals and 84% were extremely satisfied with the program. Recruitment has been successful. Participants have been diverse in terms of race/ethnicity, profession, practice characteristics and the populations they serve. Overall, participants have found the training valuable, future work will describe outcomes of the training.

